# Adenosine A_2A_ Receptors Shut Down Adenosine A_1_ Receptor-Mediated Presynaptic Inhibition to Promote Implementation of Hippocampal Long-Term Potentiation

**DOI:** 10.3390/biom13040715

**Published:** 2023-04-21

**Authors:** Cátia R. Lopes, Francisco Q. Gonçalves, Simão Olaio, Angelo R. Tomé, Rodrigo A. Cunha, João Pedro Lopes

**Affiliations:** 1 CNC-Center for Neuroscience and Cell Biology, University of Coimbra, 3004-504 Coimbra, Portugal; 2 Department of Life Sciences, Faculty of Sciences and Technology, University of Coimbra, 3004-504 Coimbra, Portugal; 3 Faculty of Medicine, University of Coimbra, 3000-534 Coimbra, Portugal

**Keywords:** adenosine, release, synapse, A_1_ receptor, A_2A_ receptor, LTP, hippocampus, crosstalk

## Abstract

Adenosine operates a modulation system fine-tuning the efficiency of synaptic transmission and plasticity through A_1_ and A_2A_ receptors (A_1_R, A_2A_R), respectively. Supramaximal activation of A_1_R can block hippocampal synaptic transmission, and the tonic engagement of A_1_R-mediated inhibition is increased with increased frequency of nerve stimulation. This is compatible with an activity-dependent increase in extracellular adenosine in hippocampal excitatory synapses, which can reach levels sufficient to block synaptic transmission. We now report that A_2A_R activation decreases A_1_R-medated inhibition of synaptic transmission, with particular relevance during high-frequency-induced long-term potentiation (LTP). Thus, whereas the A_1_R antagonist DPCPX (50 nM) was devoid of effects on LTP magnitude, the addition of an A_2A_R antagonist SCH58261 (50 nM) allowed a facilitatory effect of DPCPX on LTP to be revealed. Additionally, the activation of A_2A_R with CGS21680 (30 nM) decreased the potency of the A_1_R agonist CPA (6–60 nM) to inhibit hippocampal synaptic transmission in a manner prevented by SCH58261. These observations show that A_2A_R play a key role in dampening A_1_R during high-frequency induction of hippocampal LTP. This provides a new framework for understanding how the powerful adenosine A_1_R-mediated inhibition of excitatory transmission can be controlled to allow the implementation of hippocampal LTP.

## 1. Introduction

Adenosine is a modulator fine-tuning synaptic transmission and contributes to the encoding of the salience of information in neuronal networks [1] through the activation of inhibitory A_1_ receptors (A_1_R) and facilitatory A_2A_ receptors (A_2A_R) [2]. A_1_R are the second more abundant G-protein-coupled receptors in the brain [3] with a predominant localization in synapses, mainly presynaptically [4], in accordance with their main role of controlling excitatory synaptic transmission through the inhibition of glutamate release [5]. A_1_R are powerful inhibitors of excitatory transmission since their supramaximal activation can block excitatory synaptic transmission (e.g., [6]). The extracellular adenosine that activates A_1_R is transiently increased in excitatory synapses, even after a single transmission event [7], through a combined outflow of adenosine from presynaptic (e.g., [8]) and postsynaptic neuronal compartments (e.g., [9]) as well as upon astrocytic release of ATP and its extracellular conversion into adenosine by ecto-nucleotidases [10] to ensure heterosynaptic depression [11,12]. Overall, the A_1_R neuromodulation system is viewed as an activity-dependent feedback inhibitory system (reviewed in [3,13]), which has an increasing ability to depress excitatory synapses the more they are recruited [14].

This hypothesis that extracellular adenosine builds up with increasing recruitment of synaptic activity poses a major conceptual problem when considering particularly intense patterns of stimulation that are able to trigger long-term increases of synaptic transmission—namely, long-term potentiation, which are considered the neurophysiological basis of memory [15]. In fact, under high-frequency stimulation, one would expect a robust increase in extracellular adenosine levels that could eventually block excitatory synaptic transmission through a supramaximal activation of A_1_R. Thus, the ability of intense patterns of stimulation to trigger LTP rather than to block excitatory transmission would require a mechanism able to shut down this powerful A_1_R inhibitory system. Accordingly, it is experimentally observed that A_1_R-mediated inhibition seems to be largely limited to control LTP magnitude (e.g., [16,17]). A possible mechanism to explain this shutdown of A_1_R would be based on the previously reported ability of A_2A_R to dampen A_1_R function in brain synapses [18,19,20,21]. In fact, it has previously been reported that A_2A_R activation can decrease A_1_R binding and A_1_R-mediated inhibition of synaptic transmission [18,19]. A_2A_R are selectively engaged under conditions of high frequency recruitment of hippocampal synapses: A_2A_R antagonists are essentially devoid of effects in the control of basal synaptic transmission (e.g., [17,22,23]), whereas A_2A_R are selectively engaged to control frequency-dependent synaptic plasticity processes such as LTP in the hippocampus (e.g., [17,22,23]) as well as in other brain regions [24,25,26]. This selective engagement of A_2A_R is associated with a high frequency-induced release of synaptic ATP [8,27] coupled to its extracellular conversion into adenosine through ecto-nucleotidases [28], and the physical association of ecto-5′-nucleotidase with A_2A_R [29] ensures that synaptic ATP-derived extracellular adenosine selectively activates A_2A_R [26,30,31,32,33,34]. However, although the A_2A_R-mediated shutdown of A_1_R function is a tempting hypothesis to understand the dampening of A_1_R-mediated control of LTP, this mechanism still needs to be experimentally confirmed.

Thus, the present study was designed to test: (i) if extracellular adenosine does indeed build up in synapses according to the increased recruitment of hippocampal excitatory synapses, and (ii) if A_2A_R do indeed play a key role in dampening A_1_R during LTP induction to allow the implementation of LTP free of a putatively robust A_1_R-mediated inhibition.

## 2. Materials and Methods

### 2.1. Animals

We used adult C57bl/6j mice with 4–6 months of age of both sexes, obtained from Charles River (Barcelona, Spain). The animals were housed under controlled temperature (23 ± 2 °C) and subject to a fixed 12 h light/dark cycle, with free access to food and water. Animal procedures were performed in accordance with European Community guidelines (EU Directive 2010/63/EU) and the Portuguese law on animal care (1005/92), and they were approved by the Ethical Committee of the Center for Neuroscience and Cell Biology of Coimbra (ORBEA-128/2015). All efforts were made to reduce the number of animals used and to minimize their stress and discomfort. Thus, the animals were anesthetized in a halothane atmosphere before decapitation and, whereas the hippocampus was used in this study, other tissues from these animals were collected for use in different projects at our research center.

### 2.2. Dugs

N^6^-cyclopenthyladenosine (CPA), 8-cyclopentyl-1,3-dipropylxanthine (DPCPX), 3-{4-[2-({6-amino-9-[(2R,3R,4S,5S)-5-(ethylcarbamoyl)-3,4-dihydroxyoxolan-2-yl]-9H-purin-2-yl}amino)ethyl]phenyl}-propanoic acid (CGS21680) and 2-(2-furanyl)-7-(2-phenylethyl)-7H-pyrazolo[4,3-e][1,2,4]triazolo[1,5-c]pyrimidin-5-amine (SCH58261) were from Tocris (Bristol, UK). Adenosine and adenosine deaminase (ADA) were from Sigma (St. Louis, MO, USA). CPA, CGS21680, DPCPX and SCH58261 were made up as 5 mM stock solutions in dimethylsulfoxide (Sigma) and dilutions were prepared in artificial cerebrospinal fluid (ACSF, see constitution below), controlling for the impact of the residual amount of dimethylsulfoxide. ADA, DPCPX, SCH58261 and CGS21680 were used in supramaximal but selective concentrations: respectively, 2 U/mL [35], 50–100 nM [6], 50 nM [22,36], and 30 nM [37].

### 2.3. Adenosine Release from Mouse Hippocampal Synaptosomes

Adenosine release was assayed essentially as previously described [38]. Briefly, hippocampal synaptosomes were prepared using Percoll/sucrose gradients [39] and resuspended in Krebs–HEPES solution (in mM: NaCl 113, KCl 3, KH_2_PO_4_ 1.2, MgSO_4_ 1.2, CaCl_2_ 2.5, NaHCO_3_ 25, glucose 10, HEPES 15, pH 7.4, 37 °C). Hippocampal synaptosomes (1.2–1.4 mg protein/mL) were then incubated at 37 °C for 5 min before adding different amounts of a concentrated KCl solution (5 M) to end up with final K^+^ concentrations of 3 (control, no additionally added K^+^), 10, 15, 30 or 60 mM. After 5 min, the mixtures were centrifuged at 14,000× *g* for 10 min at 4 °C, and the supernatant was used for the analysis of the extracellular levels of adenosine. 

The separation and quantification of adenosine and its metabolites was carried out by HPLC, as previously described [40], employing a LiChroCart-RT125-4 C-18 reverse-phase column (particle size, 5 μm) combined with a UV detector set to 254 nm (Gold System, Beckman). The mobile phase was KH_2_PO_4_ (100 mM) and methanol (85/15 *v*/*v*%) at pH 6.50 with a flow rate of 1 mL/min and an injection loop volume of 50 μL. The identification and quantification of adenosine and its metabolites was achieved by calculating the peak areas then converted to concentration values (expressed as nmol/mg protein) by calibration with standards ranging from 0.1 to 50 μM.

### 2.4. Electrophysiological Recordings

Following sacrifice by decapitation, the mouse brain was quickly removed and placed in ice-cold, oxygenated (95% O_2_, 5% CO_2_), artificial cerebrospinal fluid (ACSF; in mM: 124.0 NaCl, 4.4 KCl, 1.0 Na_2_HPO_4_, 25.0 NaHCO_3_, 2.0 CaCl_2_, 1.0 MgCl_2_, 10.0 glucose). Slices (400 µm-thick) from the dorsal hippocampus were cut transverse to the long axis of the hippocampus with a McIlwain tissue chopper (Mickle Laboratory Engineering Co., Surrey, UK) and maintained for at least 1 h prior to recording in a holding chamber with oxygenated ACSF at room temperature. Slices were then transferred to a submerged recording chamber and superfused at 3 mL/min with oxygenated ACSF kept at 30.5 °C. Electrophysiological recordings of field excitatory postsynaptic potentials (fEPSP) were carried out as previously described (e.g., [41]) with the recording electrode, filled with 4 M NaCl (2–5 MΩ resistance), placed in the CA1 stratum radiatum targeting the distal dendrites of pyramidal neurons and the stimulating bipolar concentric electrode placed in the proximal CA1 stratum radiatum. Rectangular pulses of 0.1 ms were delivered every 20 s through a Grass S44 or a Grass S48 square pulse stimulator (Grass Technologies, Singapore). After amplification (ISO-80, World Precision Instruments, Singapore), the recordings were digitized (Pico ADC-42, Pico Technologies Ltd., St. Neots, UK), averaged in groups of 3, and analyzed using the WinLTP version 2.10 software [42]. The intensity of stimulation was chosen between 50–60% of maximal fEPSP response, determined on the basis of input/output curves in which the percentage of the maximum fEPSP slope was plotted versus stimulus intensity. 

Alterations of synaptic transmission were quantified as the percentage modification of the average value of the fEPSP slope taken from 15 to 20 min after beginning the application of tested drugs (DPCPX, SCH58261, adenosine, ADA, or CPA) in relation to the average value of the fEPSP slope during the 5 min that preceded the application of each tested drug. The EC_50_ value of CPA-mediated inhibition was calculated by a sigmoid fitting (variable slope and with a constant bottom value of zero) of the log concentration-response curve, as previously performed [6]. To assess the frequency-dependent effect of DPCPX, we delivered groups of 10 pulses at each tested frequency from 1 to 100 Hz first, with inter-group intervals of 10 min without stimulation (see [43]), first in the absence and then in the presence of 100 nM DPCPX. The effect of DPCPX was calculated as the ratio of the average slopes of last 5 fEPSPs in each group in the absence and in the presence of DPCPX, both normalized by the baseline calibrated with input-output curves carried out before and after DPCPX exposure. Long-term potentiation (LTP) was induced by a high-frequency stimulation train (100 Hz for 1 s). LTP was quantified as the percentage change between the average slope of the 10 fEPSPs between 50 and 60 min after LTP induction in relation to the average slope of the fEPSPs measured during the 10 min that preceded LTP induction. The effect of drugs on LTP was assessed by comparing LTP amplitude in the absence and presence of the drug in experiments carried out in different slices from the same animal. 

### 2.5. Statistical Analysis

Except for EC_50_ values that are presented as mean and 95% confidence interval, all values are presented as mean ± S.E.M. of n preparations (slices or synaptosomes from different mice). Alterations compared to baseline were estimated with a one-sample Student’s *t* test and the comparison of two experimental conditions was performed using an unpaired Student’s *t* test. Otherwise, statistical analysis was performed by one-way analysis of variance (ANOVA), followed by Tukey’s post hoc test. *p* < 0.05 was taken as threshold to consider statistical significance. Statistical analysis was performed using GraphPad Prism software (GraphPad Software, San Diego, CA, USA).

## 3. Results

### 3.1. Intensity- and Frequency-Dependent Increase of Extracellular Adenosine in Hippocampal Synapses

**Figure 1:**
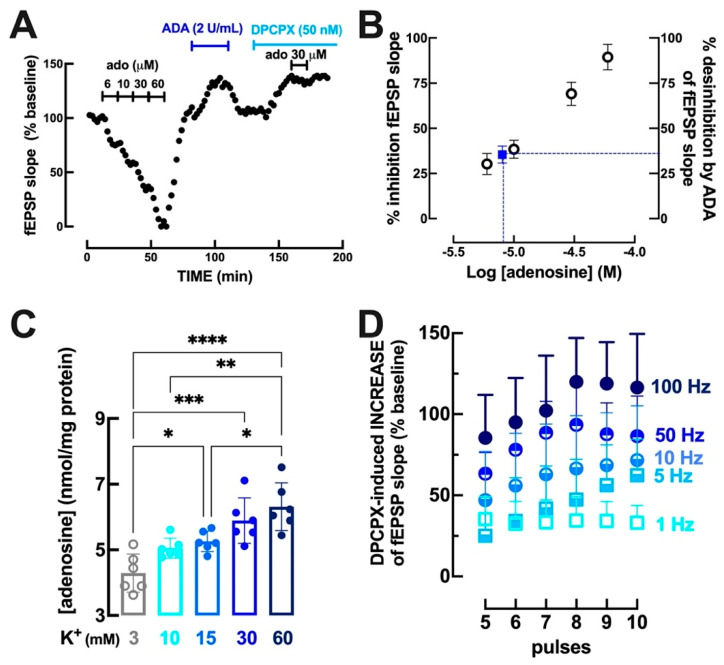
Estimation of the endogenous extracellular levels of adenosine in hippocampal synapses show that the intensity- and frequency-dependent increase in adenosine reaches levels sufficient to block synaptic transmission at higher frequencies of stimulation. (**A**) Representative experiment in a mouse hippocampal slice, showing that increasing concentrations of exogenously added adenosine (Ado, 6–60 μM) caused an increased inhibition of hippocampal synaptic transmission, measured as a decrease in the slope of field excitatory postsynaptic potentials (fEPSP) recorded in the CA1 stratum radiatum upon stimulation of the afferent Schaffer collaterals with pulses delivered at 0.1 Hz. The effect of adenosine is essentially abolished by the selective A_1_R antagonist DPCPX (50 nM) and both DPCPX and adenosine deaminase (ADA, which convert extracellular adenosine into inosine) increase fEPSP responses, implying that endogenous extracellular adenosine tonically depresses hippocampal synaptic transmission. (**B**) The interpolation of ADA-mediated disinhibition on the curve of adenosine-mediated inhibition of synaptic transmission prompts an estimate of synaptic extracellular levels of adenosine of 8 μM. Data are mean ± SEM of *n* = 6. (**C**) The mean ± SEM values (*n* = 6) of the extracellular levels of adenosine, upon incubation of mouse hippocampal synaptosomes (∼1.2–1.4 mg protein/mL) during 5 min in the presence of increasing concentrations of K^+^ (10–60 mM), showing that there is an increase in adenosine outflow proportional to the intensity of potassium-induced synaptic depolarization. * *p* < 0.05, ** *p* < 0.01, *** *p* < 0.005, **** *p* < 0.001 between indicated bars. (**D**) The effect of DPCPX on hippocampal synaptic transmission in Schaffer fibers-CA1 pyramid synapses is larger with increasing frequencies of stimulation, with pulses applied in groups of 10 with increasing frequencies from 1–100 Hz, with 10 min interval without stimulation between each group, first in the absence, then in the presence of 100 nM DPCPX. Data are mean ± SEM values (*n* = 4–6).

Adenosine caused a concentration-dependent inhibition of hippocampal synaptic transmission in the range of 6–60 μM (Figure 1A) and the highest concentration of adenosine (60 μM) nearly blocked hippocampal synaptic transmission (89.4 ± 2.9% inhibition, *n* = 6), as previously reported (e.g., [44]). As shown in Figure 1A, the removal of extracellular adenosine using adenosine deaminase (ADA 2 U/mL) caused a desinhibition of hippocampal synaptic transmission (35.4 ± 1.9% facilitation, *n* = 6), which was essentially equivalent (*p* > 0.05; unpaired Student’s *t* test) to the desinhibition caused by the selective A_1_R antagonist DPCPX (50 nM) (29.2 ± 2.6% facilitation, *n* = 6), confirming that extracellular adenosine seems to be only acting through inhibitory A_1_R under basal conditions of stimulation [17,41]. To estimate the concentration of adenosine in an active hippocampal glutamatergic synapse, the inhibitory effect of exogenously added adenosine was compared with the desinhibition caused by ADA. As illustrated in Figure 1B, the concentration of endogenous extracellular adenosine in active glutamatergic synapses under ‘basal’ stimulation (at 0.1 Hz) was estimated to be 8 µM.

We then used two parallel approaches to investigate if an increased recruitment of hippocampal synapses would trigger an increased outflow of adenosine. First, we subjected hippocampal synaptosomes (purified synapses) to increasing concentrations of K^+^ (from 10 to 60 mM), which lead to an increased depolarization of synaptosomes corresponding to a low all the way up to high intensities of stimulation of the nerve terminals (e.g., [45,46,47). As shown in Figure 1C, increasing concentrations of extracellular K^+^ triggered a progressive increase in the extracellular levels of adenosine (*p* < 0.001, one-way ANOVA), showing that the outflow of adenosine from nerve terminals increases with increasing intensities of potassium-induced synaptic depolarization. The second approach was based on the evaluation of the disinhibition of hippocampal synaptic transmission caused by blockade of A_1_R with 100 nM DPCPX, under conditions of administration of a limited number of pulses at increasing frequencies of stimulation. As summarized in Figure 1D, the disinhibition of hippocampal synaptic transmission caused by DPCPX (100 nM) was larger with increasing frequencies of stimulation (*p* < 0.001, one-way ANOVA), which is compatible with the conclusion that the synaptic levels of adenosine increase with increasing frequencies of stimulation. In fact, the average facilitation by DPCPX at 100 Hz (106.4 ± 11.1% facilitation, *n* = 5) was larger (*p* < 0.001, Tukey’s post hoc test) than at 10 Hz (62.1 ± 6.4% facilitation, *n* = 5); also, the facilitation by DPCPX at 50 Hz (83.0 ± 7.9% facilitation, *n* = 5) was larger (*p* < 0.01, Tukey’s post hoc test) than at 10 Hz; and the facilitation by DPCPX at 10 Hz was larger (*p* < 0.05, Tukey’s post hoc test) than at 1 Hz (33.9 ± 4.9% facilitation, *n* = 5).

It is striking to note that the desinhibition caused by DPCPX at the highest frequency of stimulation tested (106.4 ± 11.1% facilitation, *n* = 5, at 100 Hz) lead to an estimated concentration of endogenous extracellular adenosine in active glutamatergic synapses of over 60 µM (using the same rationale as described for Figure 1B for ‘basal’ stimulation at 0.1 Hz). Even if such an extrapolation might be unprecise, it prompts a paradoxical scenario: at frequencies used to trigger a robust long-term potentiation (LTP, with a larger number of pulses at 100 Hz), the levels of extracellular adenosine in excitatory glutamatergic synapses should be sufficient to nearly block synaptic transmission, rather than allowing a potentiation of synaptic transmission. Since more than 20 pulses at 100 Hz trigger an LTP rather than a blockade of excitatory transmission, our new data on the frequency-dependent increase in extracellular synaptic adenosine imply that A_1_R-mediated inhibition needs to be shutdown to allow high-frequency stimulation patterns to trigger an LTP.

### 3.2. A_2A_R Blockade Untethers an A_1_R-Mediated Inhibition of Hippocampal LTP

**Figure 2:**
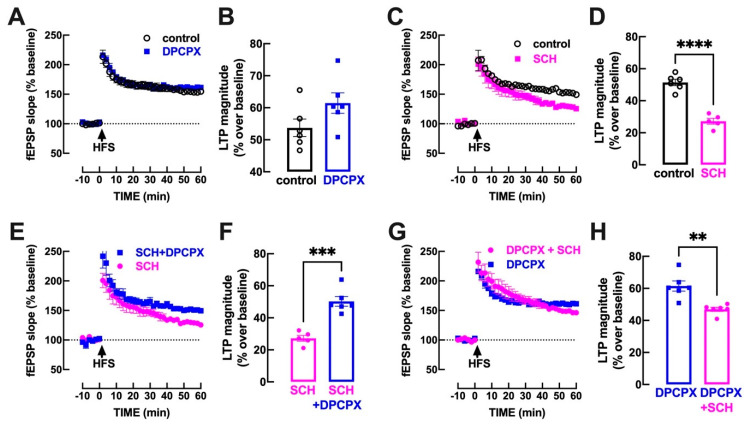
The tonic activation of A_2A_R blunts the impact of the tonic activation of A_1_R to modify hippocampal long-term potentiation (LTP). A supramaximal concentration of the A_1_R antagonist DPCPX (50 nM) did not significantly modify the amplitude of long-term potentiation (LTP) of field excitatory postsynaptic potentials (fEPSP) in Schaffer fibers-CA1 pyramid synapses of mouse hippocampal slices: the time course of fEPSP slope recordings (**A**) shows a sustained increase in fEPSP slope after delivering a high-frequency stimulation train (HFS: one train of 100 pulses at 100 Hz), which is not significantly different in the presence of 50 nM DPCPX (blue symbols) compared to its absence (black symbols), as quantified in (**B**). In contrast, the A_2A_R antagonist SCH58261 (50 nM) significantly decreased LTP magnitude (**C**,**D**). In the continuous presence of SCH58261 (50 nM), DPCPX (50 nM) was now able to significantly increase LTP amplitude (**E**,**F**). In contrast, in the continuous presence of 50 nM DPCPX, SCH58261 (50 nM) was still able to decrease LTP magnitude (**G**,**H**), albeit causing an inhibition lower than in the absence of DPCPX (**D**). Data are mean ± S.E.M. of 5–6. ** *p* < 0.01, *** *p* < 0.005, **** *p* < 0.001, Student’s *t* test.

The application of a high-frequency train (HFS) with 100 pulses at 100 Hz triggered a long-term potentiation (LTP) in Schaffer fiber-CA1 pyramid synapses of mouse hippocampal slices. As shown in Figure 2A,B, the blockade of A_1_R with 50 nM DPCPX did not significantly (*p* = 0.095; unpaired Student’s *t* test) modify the magnitude of LTP (53.7 ± 2.7% over baseline in the absence and 61.5 ± 3.2% in the presence of DPCPX, *n* = 6), confirming that A_1_R do not tonically modulate LTP in hippocampal slices of adult mice (see [16,17]). In contrast, the selective blockade of A_2A_R with 50 nM SCH58261 significantly decreased (*p* < 0.001; unpaired Student’s *t* test) the magnitude of LTP (51.4 ± 2.0% over baseline in the absence and 27.2 ± 1.9% in the presence of SCH58261, *n* = 5; Figure 2C,D), confirming that A_2A_R tonically facilitate LTP magnitude in hippocampal slices of adult mice (see [17]). Remarkably, when A_2A_R were blocked by SCH58261, it was possible to highlight a facilitatory effect (*p* < 0.001; unpaired Student’s *t* test) of DPCPX on the magnitude of LTP (in the continuous presence of SCH58261, LTP magnitude was 27.2 ± 1.9% in the absence and 50.3 ± 3.0% in the presence of DPCPX, *n* = 5; Figure 2E,F). This shows that A_2A_R blockade unveils an ability of tonic A_1_R activation to inhibit LTP magnitude. In contrast, the tonic activation of A_2A_R maintains its ability to facilitate LTP magnitude irrespective of the blockade of A_1_R: in fact, in the presence of DPCPX, SCH58261 significantly decreased (*p* < 0.001; unpaired Student’s *t* test) the magnitude of LTP (in the continuous presence of DPCPX, LTP magnitude was 61.5 ± 3.2% in the absence and 46.9 ± 1.3% in the presence of SCH58261, *n* = 6; Figure 2G,H). However, it is important to note that the amplitude of the effect of SCH58261 was lower (*p* < 0.001; unpaired Student’s *t* test) in the presence of DPCPX (23.6 ± 1.4% decrease in LTP magnitude) than in its absence (47.1 ± 3.7% decrease in LTP magnitude), which indicates that the impact of A_2A_R on LTP is a composite of an inhibition of A_1_R inhibition and a direct facilitatory effect of A_2A_R through other mechanisms (e.g., [22]).

### 3.3. A_2A_R Activation Dampens A_1_R-Mediated Inhibition of Hippocampal Transmission

**Figure 3:**
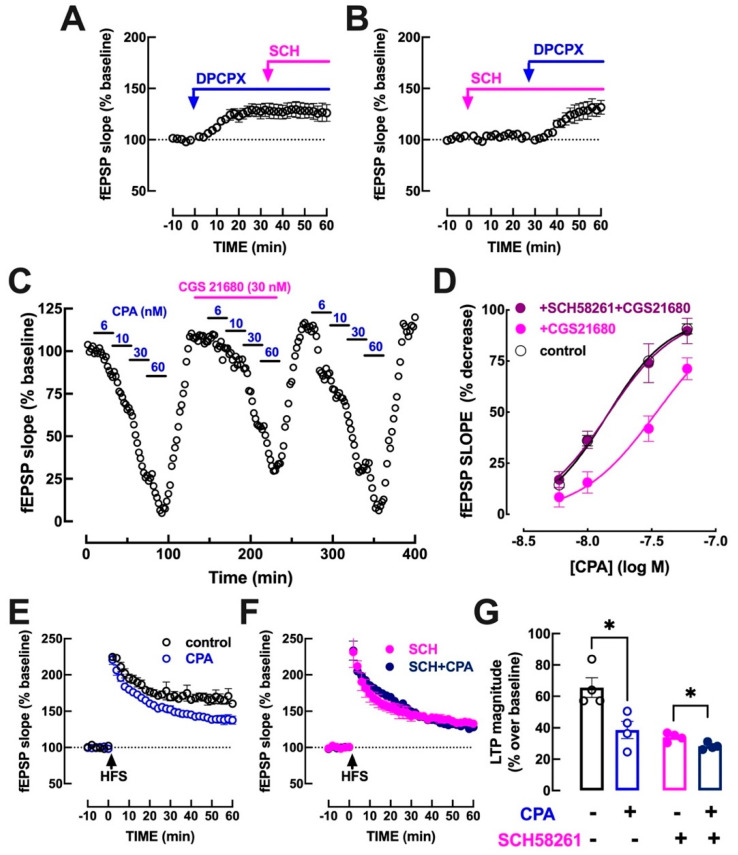
The activation of A_2A_R attenuates the ability of A_1_R to control hippocampal synaptic transmission. (**A**,**B**) A supramaximal concentration of the A_1_R antagonist DPCPX (50 nM) increased basal hippocampal synaptic transmission measured as field excitatory postsynaptic potentials (fEPSP) in Schaffer fibers-CA1 pyramid synapses of mouse hippocampal slices, irrespective of the blockade of A_2A_R with the selective antagonist SCH58261 (50 nM). (**C**) The A_2A_R agonist, CGS21680 (30 nM), attenuated the ability of the selective A_1_R agonist, CPA (6–60 nM), to inhibit basal hippocampal synaptic transmission in a manner prevented by the A_2A_R antagonist, SCH58261 (50 nM) (**D**). (**E**) CPA (30 nM) inhibited hippocampal long-term potentiation (LTP) in CA1 synapses triggered by high-frequency stimulation train (HFS: one train of 100 pulses at 100 Hz), an inhibitory effect still present but attenuated by blocking A_2A_R with SCH58261 (50 nM) (**F**), as quantified in (**G**). * *p* < 0.01, Student’s *t* test.

In contrast to the previously described ability of the tonic activation of A_2A_R to control LTP and the modulation by A_1_R of LTP, SCH58261 (50 nM) was devoid of effects on basal synaptic transmission (−1.35 ± 1.49% variation of baseline fEPSP slope alone and 1.44 ± 1.60% in the presence of DPCPX, *n* = 5, *p* > 0.05, *t*-test vs. 0%) and did not modify the disinhibition of basal synaptic transmission caused by the blockade of A_1_R by 50 nM DPCPX (28.0 ± 7.0% variation of baseline fEPSP slope by DPCPX in the absence and 28.6 ± 6.6% in the presence of SCH58261, *n* = 5, *p* > 0.05, unpaired Student’s *t* test) (Figure 3A,B).

However, the activation of A_2A_R with a supramaximal concentration of the exogenously added A_2A_R agonist, CGS21680 (30 nM; [37]), confirmed that A_2A_R activation attenuates the ability of A_1_R to inhibit synaptic transmission. In fact, as shown in Figure 3C, the selective A_1_R agonist CPA (6–60 nM) inhibited basal synaptic transmission in a concentration-dependent manner, and this inhibitory effect was attenuated in the presence of 30 nM CGS21680, which by itself did not significantly affect basal synaptic transmission (8.0 ± 4.0% variation of baseline fEPSP slope, *n* = 4, *p* > 0.05, *t*-test vs. 0%). As depicted in Figure 3D, the EC_50_ of CPA to inhibit basal synaptic transmission was 13.9 nM (95% confidence interval of 11.0–16.8 nM, *n* = 6) and it was shifted to the right in the presence of 30 nM CGS21680 (EC_50_ = 34.8 nM, 95% confidence interval of 25.8–43.8 nM, *n* = 4). Notably, this effect of CGS21680 on CPA-induced inhibition was eliminated in the presence of 50 nM SCH58261 (EC_50_ = 15.3 nM, 95% confidence interval of 9.7–20.8 nM, *n* = 4; Figure 3D), showing that the effect of CGS21680 is due to A_2A_R activation. SCH58261 (50 nM) did not affect the CPA-induced inhibition of basal synaptic transmission (EC_50_ = 14.3 nM, 95% confidence interval of 9.9–18.6 nM, *n* = 4). Finally, the time course experiment depicted in Figure 3C shows that this A_2A_R-induced attenuation of A_1_R-mediated inhibition disappeared upon the washing out of CGS21680 (*n* = 2), suggesting that it may be a short-lived inhibition dependent on the ongoing activation of A_2A_R. 

Finally, we tested the impact of the exogenous activation of A_1_R on hippocampal LTP. Always compared to baseline, CPA (30 nM) alone decreased LTP magnitude by 41.0 ± 7.0% (65.5 ± 6.3% over baseline in the absence and 38.7 ± 5.5% in the presence of CPA, *n* = 4, *p* = 0.018 < 0.05, unpaired Student’s *t* test) (Figure 3E,G), whereas in the presence of 50 nM SCH58261, CPA (30 nM) caused a quantitatively lower (16.9 ± 0.9%, *n* = 4) inhibitory effect (in the continuous presence of SCH58261, LTP magnitude was 34.04 ± 1.4% over baseline in the absence and 28.3 ± 1.1% in the presence of CPA, *n* = 4, *p* = 0.016, unpaired Student’s *t* test) (Figure 3F,G). This prompts two tentative conclusions: (i) the unleashing of a robust A_1_R tonic inhibition upon the A_2A_R blockade by endogenous adenosine (see Figure 2) forces a ceiling-like effect for A_1_R-mediated inhibition, which limits the additional inhibitory impact of the exogenous activation of A_1_R with CPA; (ii) since A_1_R only inhibited LTP (maximally by circa 60%) whereas A_1_R nearly blocked basal synaptic transmission (e.g., Figure 1A and Figure 3C), there might be other mechanisms, apart from the presently described ability of A_2A_R activation, to restrain A_1_R-mediated inhibition of hippocampal excitatory transmission during LTP induction. 

## 4. Discussion

The present study provides evidence supporting a new framework for understanding how the powerful adenosine A_1_R-mediated inhibition of excitatory transmission can be controlled to allow the implementation of long-term increases of synaptic transmission (LTP) upon high-frequency stimulation. Thus, in spite of the increased synaptic levels of adenosine triggered by high frequency trains, the selective engagement of A_2A_R during these high frequency trains decreases the efficiency of the A_1_R-mediated inhibition to allow the implementation of LTP. This conclusion was based on two parallel and concurrent pieces of evidence: (i) the activation of A_2A_R decreases the efficiency of A_1_R activation to inhibit excitatory synaptic transmission; (ii) the blockade of A_2A_R during LTP revealed an ability of A_1_R to control LTP magnitude, which was absent under conditions of basal synaptic transmission.

The presently described ability of A_2A_R to shut-down A_1_R-mediated inhibition is selectively engaged during LTP induction. As previously demonstrated, this likely results from the increased ATP release during high frequency stimulation [8,27] associated with the CD73-mediated formation of ATP-derived adenosine that selectively activates synaptic A_2A_R during LTP [22,26,31,32,33,34]. In fact, SCH58261 was devoid of effects during basal synaptic transmission (see Figure 3) while it decreased LTP magnitude, as previously reported in these and other brain synapses (reviewed in [48]). The impact of A_2A_R on LTP is expected to involve at least two parallel mechanisms: (i) on one hand, there is an ability of A_2A_R to engage p38 and Src kinases to increase synaptic function [22,49,50]; (ii) on the other hand, the facilitatory effect of A_2A_R on LTP might also result from the presently described attenuation of inhibitory A_1_R-mediated effects in excitatory synapses. The observed effect of SCH58261 in the presence of DPCPX (Figure 2G,H) clearly indicates that A_2A_R can directly control LTP magnitude independently of A_1_R. However, the observed lower amplitude of the effect of SCH58261 in the presence (Figure 2G,H) compared to absence of DPCPX (Figure 2C,D) also shows that there is a contribution of the A_2A_R-mediated control of A_1_R for the control of LTP magnitude by A_2A_R. 

The mechanism operated by A_2A_R to dampen A_1_R function in hippocampal synapses still needs to be characterized. Previous studies in striatal synapses indicated that A_1_R and A_2A_R form heteromeric complexes, where A_2A_R inhibit A_1_R-mediated signaling [20]; however, the formation of A_1_R-A_2A_R heteromers has not been documented in hippocampal synapses. It has been demonstrated that A_1_R and A_2A_R are co-localized in glutamatergic terminals of the hippocampus [51], but the sole existence of an A_1_R-A_2A_R heteromeric mechanism underlying the synaptic shut-down of A_1_R by A_2A_R is somewhat difficult to reconcile with the far more abundant density of synaptic A_1_R compared to the circa 20-times lower density of A_2A_R in hippocampal synapses (cf. [4,52]). An alternative that may enable this quantitative discrepancy of A_1_R versus A_2A_R densities in hippocampal synapses to be overcome might be to conceive that A_1_R function in synapses is controlled by the engagement of the A_2A_R transducing system. The traditional view is that A_1_R and A_2A_R are oppositely coupled to the activity of adenylcyclase-cAMP-protein kinase A pathway, but the evidence to support the involvement of this canonical pathway in the control by A_1_R and A_2A_R of excitatory synaptic transmission still needs to be provided (reviewed in [2]). Clearly, the definition of the different transducing systems operated by synaptic A_1_R and A_2A_R will be required prior to the further probing of this hypothesis that the engagement of A_2A_R-induced transduction system may be responsible for controlling the coupling of A_1_R with G-proteins and/or the intracellular or intra-membrane transducing systems operated by A_1_R to depress excitatory synaptic transmission. The latter also still need to be experimentally defined. Additionally, the question of whether this A_2A_R-mediated control of A_1_R function is mostly taking place presynaptically to control glutamate release or postsynaptically to control the responsiveness to glutamate will need to be determined. In fact, both A_1_R and A_2A_R are located presynaptically and postsynaptically in hippocampal synapses [4,52], and there is previous evidence showing that A_1_R [5] and A_2A_R can affect excitatory transmission in the hippocampus by both presynaptic and postsynaptic effects [19,22,53,54]. 

The ability reported here of A_2A_R to shut down A_1_R-mediated inhibition during high-frequency stimulation in order to allow the implementation of LTP also prompts questions regarding whether the engagement of A_2A_R upon high-frequency stimulation might also shut down other inhibitory modulation systems in synapses. In fact, apart from A_1_R, there are other synaptic inhibitory modulation systems that are also expected to be recruited in an activity-dependent manner, in particular the cannabinoid CB_1_R system. CB_1_R are also located in hippocampal excitatory synapses [55], and their activation can depress excitatory synaptic transmission as well as LTP in the hippocampus (e.g., [56]). Interestingly, A_2A_R can control CB_1_R-mediated inhibition (e.g., [57,58]), namely, in hippocampal synapses [59,60], and it remains to be tested whether an eventual A_2A_R-induced down-regulation of CB_1_R-mediated inhibition might also contribute to LTP induction. The same rationale might apply for GABA_B_R or P_2Y1_R that inhibit hippocampal glutamate synapses (e.g., [61,62]), with the latter shown to be modulated by A_2A_R in astrocytes [63].

It is interesting to note that the comparison of the impact on A_1_R function of endogenously activated A_2A_R with the exogenous pharmacological activation of A_2A_R revealed some unexpected differences. In fact, the exposure to CGS21680, expected to supramaximally activate A_2A_R, appears to be less efficacious to modulate A_1_R function (Figure 3C) when compared to the exposure of SCH58261 (Figure 2F and Figure 3G), which only reveals the effects of endogenously activated A_2A_R. This might be associated with the previous description of adenosine receptors in the hippocampus with an atypical pharmacological profile [64,65,66], leading to the possibility that CGS21680 might also directly interact with A_1_R in the hippocampus (e.g., [66]). Another factor contributing to the apparently greater efficiency of the endogenous versus exogenous activation of A_2A_R to control A_1_R function and LTP magnitude is the existence of different pools of A_2A_R with different and, often, opposite effects, as illustrated by the different impact of neuronal and astrocytic A_2A_R in the hippocampus [67], the presynaptic and postsynaptic A_2A_R in the striatum [68,69], or A_2A_R in different brain regions (cf. [24,70]). In fact, upon LTP, there is a predominant activation of A_2A_R in forebrain neurons by ATP-derived extracellular adenosine as a result of an increased synaptic release of ATP (e.g., [22,31,32]); in contrast, when applying CGS21680, all different populations of A_2A_R will be pharmacologically engaged, namely, some populations of A_2A_R that may not be physiologically recruited during LTP. In this sense, it is unwise to directly compare the effects of endogenously activated A_2A_R (indirectly assessed by the effects of SCH58261) and the effects of exogenous activation of A_2A_R with CGS21680.

This mechanism of A_2A_R-mediated downregulation of A_1_R function is selectively engaged under LTP-like conditions and is not operating at other firing patterns that are not associated with the activation of A_2A_R. This reinforces our previous contention that A_1_R function is a low pass filter to limit basal synaptic transmission, which likely increases its functional impact with increasing intensities of synaptic recruitment until A_2A_R are engaged to turn off this gating system. In fact, A_1_R are conceptualized as a feedback homeostatic mechanism to restrain excessive excitatory synaptic transmission (reviewed in [3]). The A_1_R inhibitory system is proposed (see [13]) to be a common operator of autocrine control of synaptic activity with feedback presynaptic inhibition upon increased outflow of adenosine as a result of postsynaptic activation [9,71,72] and, also, paracrine control in the circuit through processes of heterosynaptic depression involving the outflow of adenosine from astrocytes [10] or microglia [73]. The A_1_R-mediated negative-feedback mechanism is engaged even after a single stimulation pulse [7], and it maintains its efficiency over a wide dynamic range in neocortical synapses [14] as well as in hippocampal synapses (see Figure 1D), namely in conditions of mild induction of synaptic potentiation that might be not be sufficient to not engage A_2A_R, such as upon metabolic imbalance- [74], NO- [16], chemically- [75], or theta burst-induced potentiation [76,77]. In contrast, upon high-frequency stimulation, A_1_R have a limited role in the control of LTP magnitude, as also previously observed using a pharmacological blockade of A_1_R [16,17] or upon genetic deletion of A_1_R [78]. Importantly, when A_2A_R are blocked, thus eliminating the constraint on A_1_R function, it was found that A_1_R were now able to robustly inhibit LTP magnitude (Figure 2E,F). Interestingly, there was an apparently lower efficacy of A_1_R to inhibit LTP (near 70% maximal inhibition) compared to the near blockade of basal synaptic transmission, even upon the blockade of A_2A_R. This may be for two reasons, which should be experimentally explored in future studies: (i) the different mechanism(s) operated by A_1_R to inhibit synaptic transmission (e.g., [5]) become less efficient under conditions of LTP; (ii) other synaptic modulation systems, apart from A_2A_R, are engaged during LTP to decrease A_1_R-mediated inhibition of excitatory transmission. 

The ability of selectively engaging A_2A_R to control A_1_R function to format the magnitude of hippocampal LTP that was demonstrated here is strictly dependent on the dynamics of the extracellular levels of purines, specifically within excitatory synapses, where these processes are taking place. We have now confirmed in hippocampal synapses that there is an activity-dependent increase in A_1_R-mediated inhibition of hippocampal synaptic transmission with increasing frequencies of stimulation (Figure 1D), consistent with an activity-dependent build-up of extracellular adenosine within excitatory synapses of the hippocampus, as previously shown to occur in the neocortex [14]. This increased outflow of adenosine with increasing intensities of nerve stimulation was further confirmed in synaptosomes (Figure 1C), identifying the presynaptic compartment as an additional source of activity-dependent outflow of extracellular adenosine. We did not investigate the mechanism of this synaptic outflow of adenosine, which has previously been suggested to involve a release of adenosine through exocytosis [79] or through equilibrative nucleoside transporters [8,71,72] as well as an ATP-derived formation of extracellular adenosine through the ecto-nucleotidase pathway [8]. Instead, we attempted to indirectly estimate the synaptic ‘concentration’ of adenosine based on its synaptic effects, and we reached an estimate of 8 µM under basal stimulation conditions and of over 60 µM upon high-frequency stimulation. These values are one to two orders of magnitude higher than these previously reported using several elegant techniques, such as microdialysis (0.04–0.9 µM; [80,81]), enzyme-based sensors (0.25–1 µM; [71,74,82]), voltammetry-based electrodes (0.2 µM; [83]), or adenosine-sensor cells (0.1 µM; [84]). Since the size of the devices used in all of these elegant methods to detect transient variations of the extracellular levels of adenosine only allows quantification of the levels of extracellular adenosine outside synapses, the present estimates indicate the existence of a strong gradient of extracellular adenosine within and surrounding excitatory synapses. The existence of such spatial gradients of extracellular adenosine makes it illogical that such a thing as an extracellular ‘concentration’ of adenosine might exist. Importantly, this gradient is expected to vary dynamically according to the pattern of activity to allow adenosine to fulfil its different roles both as a modulator within synapses and as a neuron-glia signal also outside synapses (reviewed in [13]). Novel experimental strategies to directly monitor transient oscillations of synaptic and peri-synaptic adenosine will be required to clarify the relation between the spatiotemporal pattern of the extracellular levels of adenosine with the recruitment of the different adenosine receptors. Furthermore, given that a component of extracellular adenosine originating from CD73-mediated ATP-derived adenosine [22,26,31,32,33,34] is critical for A_2A_R activation and formats the status of A_1_R function, and since this ATP release is also pulsatile and transient according to the pattern of synaptic stimulation [8,26,27,85], comprehension of the dynamic function of the adenosine neuromodulation system will require the clarification of the spatiotemporal dynamics of both extracellular ATP and adenosine within different synapses.

## 5. Conclusions

The present study identifies the engagement of A_2A_R during high frequency-induced LTP as a key mechanism to shut-down the robust A_1_R-mediated inhibition engaged by the activity-dependent increase of extracellular adenosine in excitatory hippocampal synapses.

## Data Availability

The data that support the findings of this study are available from the corresponding author upon reasonable request.

## Abbreviations

A_1_R: adenosine A_1_ receptors; A_2A_R: adenosine A_2A_ receptors; ACSF: artificial cerebrospinal fluid; ADA: adenosine deaminase; CPA: N^6^-cyclopenthyladenosine; DPCPX: 1,3-dipropyl-8-cyclopentylxanthine; EPSP: excitatory postsynaptic potential; LTP: long-term potentiation; SCH58261: 2-(2-furanyl)-7-(2-phenylethyl)-7H-pyrazolo[4,3-e][1,2,4]triazolo[1,5-c]pyrimidin-5-amine.
